# Protective Effect of Polyoxometalates in {Mo_132_}/Maghemite Binary Superlattices Under Annealing

**DOI:** 10.3389/fchem.2019.00830

**Published:** 2019-11-29

**Authors:** Romain Breitwieser, Adrien Garnier, Thomas Auvray, Anh-Tu Ngo, Benoit Baptiste, Nicolas Menguy, Anna Proust, Christophe Petit, Florence Volatron, Caroline Salzemann

**Affiliations:** ^1^Laboratory MONARIS, Sorbonne Université, CNRS UMR8233, Paris, France; ^2^Laboratory IPCM, Sorbonne Université, CNRS UMR8232, Paris, France; ^3^Laboratory IMPMC, Sorbonne Université, CNRS UMR7590, Paris, France

**Keywords:** binary superlattices, maghemite, polyoxometalates, magnetism, annealing

## Abstract

The binary assembly DDA-{Mo_132_}/OA-γ-Fe_2_O_3_ (DDA = didodecyldimethylammonium, {Mo_132_} = [Mo_132_O_372_(CH_3_COO)_30_(H_2_O)_72_]^42−^, OA = oleic acid) constitutes one of the two examples in the literature of binary superlattices made of a mixing of nanocrystals and oxo-clusters. In a precedent work, we reported in details the preparation of such magnetic binary systems and studied the effect of the nature of the polyoxometalates (POMs) on the magnetic properties. In the present paper, we study the stability of this model binary assembly under heating at various temperatures. Indeed, especially if magnetic and/or transport properties are targeted, an annealing can be essential to change the phase of the nanocrystals in a more magnetic one and/or to desorb the organic capping of the nano-objects that can constitute an obstacle to the electronic communication between the nano-objects. We gave evidence that the maghemite organization in the binary assembly is maintained until 370°C under vacuum thanks to the presence of the POMs. This latter evolve in the phase MoO_3_, but still permits to avoid the aggregation of the nanocrystals as well as preserve their periodical arrangement. On the contrary, an assembly made of pure γ-Fe_2_O_3_ nanocrystals displays a clear aggregation of the nano-objects from 370°C, as attested by transmission and scanning electronic microscopies and confirmed by magnetic measurements. The stability of the magnetic nanocrystals in such POMs/nanocrystals assemblies opens the way to (i) the elaboration of new binary assemblies from POMs and numerous kinds of nanocrystals with a good control on the magnetic properties and to (ii) the investigation of new physical properties as exchange coupling, or magneto-transport in such systems.

## Introduction

Binary superlattices are made of periodical arrangement of nano-objects of different nature (metallic or semi-conducting nanocrystals, micelles, clusters…), size and shape (Kiely et al., 1998; Redl et al., 2003; Rogach, 2004; Talapin, 2008; Vanmaekelbergh, 2011). The infinity of possible assemblies as well as the fine control of the stoichiometry open the way to the development of new binary systems with highly controlled emergent properties and a great versatility (Claridge et al., 2009; Bodnarchuk et al., 2013; Ye et al., 2013b, 2015; Lu et al., 2015; Paik et al., 2015; Yang et al., 2015; Breitwieser et al., 2016; Künzle et al., 2016; Lim et al., 2017; Udayabhaskararao et al., 2017). Up to now several systems reported in the literature have shown crucial properties directly correlated to the binary assembly in various application domains such as magnetism, transport, optics, and catalysis (Urban et al., 2007; Chen et al., 2010; Dong et al., 2010; Ko et al., 2010; Kang et al., 2013a,b; Ye et al., 2013a; Breitwieser et al., 2016; Wu et al., 2017).

Beyond obtaining a binary system with specific properties, one issue can be crucial, specifically in the magnetism and transport fields: the annealing of the binary assembly. Indeed, an annealing can be necessary to change the phase of the nanocrystals and form hard magnetic phases (Zeng et al., 2002; Lisiecki et al., 2007; Parker et al., 2007; Demortière and Petit, 2011; Dong et al., 2011), or to desorb the organic capping around the nano-objects that can be an obstacle to transport measurements (Chen et al., 2013). As the surrounding ligand has a key role in the formation of the binary superlattices (Boles and Talapin, 2015; Wei et al., 2015; Huang et al., 2016; Travesset, 2017), the ligand desorption has to be done after the formation of the binary assembly. However, due to the needed high temperatures, the annealing procedure can induce coalescence phenomena leading to an increase in the size of the NCs, a modification of the shape and a change in the organization (Klemmer et al., 2003; Alloyeau et al., 2010; Demortière and Petit, 2011). In this context, one of the main challenges is to be able to anneal the nanostructured film while preventing from the aggregation of the nano-objects and keeping the periodicity in the binary system, both for addressing individual magnetic objects and for controlling the charge transport (Dong et al., 2011; Altantzis et al., 2016).

Some years ago, we reported one of the two examples in the literature consisting in the binary assembly of two kinds of nano-objects with drastic different natures : nanocrystals and oxo-clusters. We described the formation of DDA-{Mo_132_}/OA-γ-Fe_2_O_3_ (DDA = didodecyldimethylammonium; OA = oleic acid) binary superlattices made of the assembly of polyoxometalates (POMs) and maghemite nanocrystals (MNCs) (Breitwieser et al., 2016). By a judicious choice of the ligand, we showed a fine-tuning of the magnetic properties of the MNCs film. In this article, we further characterize this DDA-{Mo_132_}/OA-γ-Fe_2_O_3_ model system and study its stability after annealing at high temperature by trying to investigate three crucial issues : (i) the preservation of the periodical arrangement; (ii) the assessment of the highest temperature of annealing before collapsing the system; (iii) the intrinsic stability of the nano-objects constitutive of the binary assembly.

## Materials and Methods

### Chemicals

For the synthesis of oleic acid-capped γ-Fe_2_O_3_, iron chloride (FeCl_3_.6H_2_O, 99%) was purchased from Prolabo, sodium oleate (C_18_H_33_NaO_2_, 97%) from TCI, oleic acid (90%) and 1-octadecene (90%) from Sigma-Aldrich. For the synthesis of DDA-Mo_132_, the precursor (NH_4_)_42_[Mo_132_O_372_(CH_3_COO)_30_(H_2_O)_72_]·300H_2_O·10CH_3_COONH_4_ (NH_4_-Mo_132_) was synthesized as described in the literature (Müller et al., 1998) and the DDA and all solvents were purchased from Sigma-Aldrich.

### Synthesis of the Nano-Objects

γ-Fe_2_O_3_ nanocrystals were made by slightly modifying the synthesis reported by Park et al. (2004) as described in our precedent article (Breitwieser et al., 2016). In particular, 1-octadecene was used (instead of 1-hexadecene) in order to obtain ~7.7 nm iron oxide nanocrystals. Briefly, the iron-oleate complex was first prepared then dissolved in a mixture of 1-octadecene and oleic acid at room temperature. This mixture was heated to the boiling point of 1-octadecene (b.p. 317°C) under vigorous stirring and was refluxed for 30 min and then cooled to room temperature giving rise to a black gel containing the nanocrystals. The nanocrystals were separated and washed four times with a large excess of ethanol. After four washings 40 μL of oleic acid (OA) are added in a volume ratio of 1:75. The nanocrystals were finally dispersed in chloroform to give an oily ferrofluid.

The DDA-Mo_132_ was prepared by counter-cation exchange of the NH_4_-Mo_132_ precursor as described before (Breitwieser et al., 2016) by stirring the NH_4_-Mo_132_ precursor in an excess of DDA in a water-chloroform biphasic solution.

### Samples Preparation

#### Binary Superlattices

Binary superlattices were obtained as detailed before (Breitwieser et al., 2016). Briefly, colloidal binary solutions were prepared from equal volumetric mixtures of alkylammonium-{POMs} and oleic acid-capped maghemite MNCs dispersed in CHCl_3_. Supported coassemblies were then obtained using a dip method by drying the mixture directly on horizontally immersed highly ordered pyrolytic graphite (HOPG) or amorphous carbon TEM grids at controlled temperature and in a CHCl_3_ saturated atmosphere. The volume of binary solution was deposited according to the substrates size. The TEM grids were immersed in 20 to 30 μL giving ~1–10 MNCs layers. HOPG substrates of 10 × 5 mm^2^ were immersed in 200 to 800 μL giving thickness from dozen to hundred MNCs layers. It is worth mentioning that samples made from the binary mixture were accompanied with reference samples of pure maghemite nanocrystals grown in identical conditions for better comparison.

#### Thermal Treatment

The HOPG or the copper grid modified by the binary film was introduced in a furnace (NEYTECH Qex) connected to a vacuum pump. The targeted temperature was reached with a ramp of 10°C/min then the sample was left at this temperature during 1 h under vacuum. The powder of POMs was ground then placed in a crucible to be subjected to exactly the same treatment.

### Samples Characterization

A 100~kV transmission electron microscope (TEM, JEOL JEM-1011) was used to characterize both maghemite and binary assemblies.

Conventional TEM (Bright field and high resolution), high-angle annular dark field imaging in scanning transmission electron microscope mode (STEM-HAADF) and X-ray energy-dispersive spectroscopy elemental mapping (STEM-XEDS) experiments were performed on a JEOL-2100F microscope installed at IMPMC (Paris, France), operating at 200 kV, equipped with a field emission gun, a JEOL detector with an ultrathin window allowing detection of light elements and a scanning TEM (STEM) device, which allows Z-contrast imaging in HAADF mode.

Imaging of the reference and binary samples deposited on HOPG were performed with a SU-70 Hitachi FEG-SEM.

Hysteresis loops at 3 K and thermal dependence of the γ-Fe_2_O_3_ magnetization (3–300K) were measured with a commercial SQUID magnetometer (Cryogenic S600) with applied fields up to 4 T.

To characterize the POMs powder, thermogravimetric analysis (TGA) was performed under N_2_ between 20 and 600°C with a SDT-Q600 device. IR spectra were recorded from a KBr pellet on a Jasco FT/IR 4100 spectrometer. The *in-situ* X-ray powder diffraction (XRPD) patterns were recorded at the X-ray diffraction platform of the IMPMC using an X'Pert Pro MPD Panalytical diffractometer equipped with a Co Kα radiation source (λKα_1_ = 1.78897 Å, λKα_2_ = 1.79285 Å), an X'Celerator detector. The sample was gently ground and placed on a Si zero background holder. Then the experiment was performed in Bragg Brentano geometry at 293 K.

## Results and Discussion

### Native Superlattices

**Figure 1:**
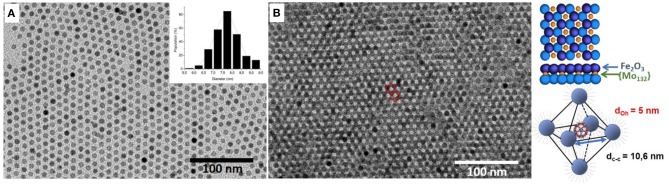
TEM images of **(A)** 7.7 nm Maghemite (γ-Fe_2_O_3_) nanocrystals and of **(B)** γ-Fe_2_O_3_ NCs/{Mo_132_} POMs binary superlattice. Schematic representation of the octahedral insertion of the POMs in the nanocrystal network.

In this study we considered 7.7 ± 0.50 nm Maghemite (γ-Fe_2_O_3_) nanocrystals synthesized by thermal decomposition of iron chloride at high temperature by following a method (Ngo et al., 2013) similar to that reported by Park et al. (2004) (see Experimental section). Their low size dispersion (~9%) favors their self-assembly in a hexagonal network characterized by a center-to-center inter-particle distance (d_cc_) equal to 11.0 ± 0.5 nm (Figure 1A and Supplementary Figure 1). Such large distances show that the maghemite film is not compact. This is explained by the excess of oleic acid added at the end of the procedure and necessary to obtain the ordered film (see Experimental section). Their association with Keplerate [Mo_132_O_372_(CH_3_COO)_30_(H_2_O)_72_]^42−^ ({Mo_132_}) POMs induces the formation of binary superlattices (Breitwieser et al., 2016). The suitable C_60_-like hollow spherical POM shape (with icosahedral symmetry (Müller et al., 1998), its large size (2.9 nm) and the use of hydrophobic long alkyl chains as counter-cations that confer them core-shell structure and solubility properties similar to those of MNCs favor the co-cristallization (Breitwieser et al., 2016). Supported binary assemblies were then obtained upon solvent evaporation either on immersed amorphous carbon coated TEM grid or HOPG. The TEM images exhibit typical [111]-projected hexagonal lattices of nanocrystal bilayers showing large domains (>500 nm^2^) in which the presence of DDA-{Mo_132_} can be observed in the octahedral interstices of the hexagonal MNCs lattice (Figure 1B). The center-to-center inter-particle distances between the MNCs in the binary superlattices are equal to 11.0 ± 0.5 nm, similar to that of the pure maghemite organization. This confirms that the POM is small enough to insert in the octahedral interstices without changing the MNCs inter-particle distance.

**Figure 2:**
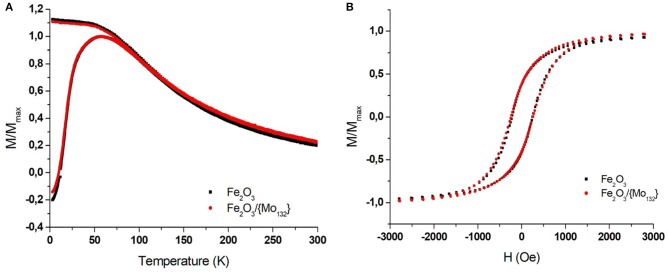
SQUID measurements of γ-Fe_2_O_3_ magnetization for a MNCs thin film (Black squares) and -γ-Fe_2_O/{Mo_132_} binary thin film (red circles). **(A)** The temperature-dependence FC and ZFC measured with in-plan field of 100 Oe and **(B)** the in-plan field-dependence (measured at 3 K).

**Figure 3:**
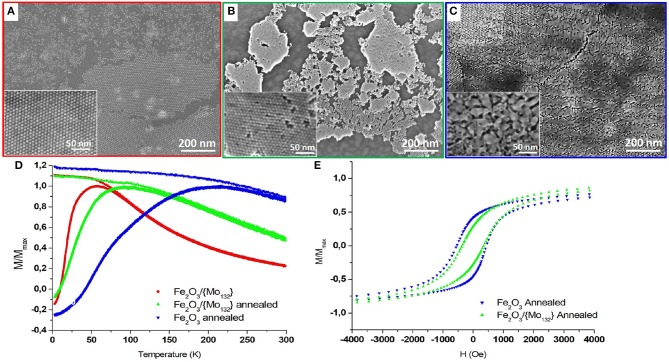
FEG-SEM images of a γ-Fe_2_O_3_/ Mo_132_ binary superlattice on HOPG **(A)** before and **(B)** after annealing at 370°C for 1 h under vacuum and of **(C)** Annealed γ-Fe_2_O_3_ film. The corresponding SQUID measurements **(D)**. The temperature-dependence FC and ZFC measured with in-plan field of 100 Oe and **(E)** the in-plan field-dependence (measured at 3 K) of γ-Fe_2_O_3_ γ-Fe_2_O_3_/{Mo_132_} binary thin film before (red circles) and after (green triangle up) annealing and of annealed γ-Fe_2_O_3_ film (blue triangle down).

The magnetic properties of the films (pure Maghemite and DDA-{Mo_132_}/OA-γ-Fe_2_O_3_ binary assemblies) have been characterized using a superconducting quantum interference device (SQUID) magnetometer. The superparamagnetic behavior of the MNCs was probed by performing field-cooling/zero-field cooling (FC/ZFC) measurements of magnetization vs. temperature (3–300 K) in an in-plane field of 100 Oe. The ferromagnetic-superparamagnetic (i.e., blocked-unblocked) transition can be determined by the temperature dependence of the magnetization that exhibits a maximum in ZFC curves. It is important to mention that the corresponding temperature (called blocking temperature, *T*_*B*_) depends on both the MNCs volume (magnetic anisotropy) and on their dipolar interactions. For isolated MNCs (no interaction), the blocking temperature is directly related to the anisotropy magnetic energy (K_eff_V) of the material, where K_eff_ is the anisotropic constant (that depends on the surface, the crystallinity and the shape) and V the volume of the MNCs. When we consider assemblies of magnetic NCs, they are no longer isolated and the presence of dipole-dipole interactions induces an interaction field (Dormann et al., 1998). The magnetic energy of a nanoparticle interacting with its neighbors, corresponding to the barrier energy (E_b_), will then be written: $E_{b}=K_{eff}V-{\;J}_{eff}\vec{M}\left(T\right).\;\langle \vec{M}\;\left(T\right)\rangle$ where J_eff_ is the exchange coupling constant, $\vec{M}\left(T\right)$ is the magnetization vector of the sublattice and $J_{eff}\;\langle \vec{M}\;\left(T\right)\rangle$ represents an effective interaction mean field acting on $\vec{M}\left(T\right)$ (Mørup et al., 2010). It appears that the dipolar interaction energy is directional and will depend on the geometrical arrangement of the MNCs in the assembly (Kechrakos and Trohidou, 1998; Mørup et al., 2010). The isolated 7.7 ± 0.70 nm Maghemite (γ-Fe_2_O_3_) nanocrystals are characterized by a blocking temperature (T_B_) equal to 55 K while it is 57 K in the film (Figure 2A, black squares). This latter value, very close to that of isolated MNCs, confirms the formation of a non-compact film characterized by very low interaction between MNCs due to the presence of oleic acid excess. The FEG-SEM images of the binary superlattice show long-range ordered domains of DDA-{Mo_132_}/OA-γ-Fe_2_O_3_ MNCs on HOPG (Figure 3A). As the presence of the POMs do not induce any change in the inter-particle distances in the MNCs network, the binary superlattice exhibits the same magnetic properties as the maghemite film ones with a T_B_ equal to 57 K (Figure 2A, red circles). The hysteresis loops (magnetization vs. applied magnetic field) show the same ferromagnetic behavior at 3 K with a coercitivity *H*_*c*_ equal to 230 Oe for both samples (Figure 2B). As discussed in the introduction, one important challenge in this domain concerns the annealing of the film that can bring important improvements in magnetic and/or transport properties. The main difficulty of this treatment relies on the possibility to keep the periodicity and avoid the aggregation of the NCs in the binary superlattice. To further characterize the DDA-{Mo_132_}/OA-γ-Fe_2_O_3_ MNCs binary system, we studied its stability under annealing at various temperatures.

### Annealing at 370°C

The previous samples (Maghemite and DDA-{Mo_132_}/OA-γ-Fe_2_O_3_ binary assemblies) are both annealed at 370°C for 1 h under vacuum (see experimental section). The effect of annealing for the reference sample (maghemite film) and for the binary superlattice results in clear differences on the film morphology and on their corresponding magnetic properties (Figure 3).

SEM-FEG images of the annealed binary superlattice give evidence that the integrity of the organization is maintained despite a drastic decrease of the d_cc_ inter-particle distance from 11.0 to 8.9 ± 0.50 nm after annealing (Figure 3B). Conversely, the annealed OA-γ-Fe_2_O_3_ (reference sample) exhibits a high coalescence of the MNCs in surface, while the organization is preserved in the bulk (Figure 3C). Indeed, compact underlying layers of organized MNCs are observed, characterized by an inter-particle distance d_CC_ equal to 9.6 ± 0.50 nm. This coalescence between MNCs has a strong influence on their magnetic properties. Indeed, a drastic increase of both the T_B_ (ΔT_B_ = 159 K) from 57 to 216 K (Figure 3D, blue down triangles) and the coercive field (ΔHc = 307 Oe) from 230 to 537 Oe (Figure 3E, blue down triangles) are observed. The ZFC curve is relatively large with a shoulder at 75 K. Such magnetic behaviors can be explained by the presence of coalesced MNCs leading to different sizes. The resulting widening of the size distribution causes the increase of the distribution of barrier energy E_b_ according to two aspects. On the one hand, the bigger MNCs (coalesced) are characterized by higher magnetic anisotropy (K_eff_V) compared to that of 7.7 nm MNCs. Hence, the distribution of size will induce an increase of the distribution of the magnetic anisotropy energy of the MNCs. On the other hand, the size distribution associated to the decrease of the interparticle distance and the inhomogeneity of the film will contribute to an increase in the dipolar magnetic interactions and an inhomogeneous interaction field. The shoulder in the ZFC curve (100 Oe) at 75 K illustrates these effects. It corresponds to the magnetic response of the underlying film of non-coalesced 7.7 nm MNCs observed on the SEM-FEG images (Figure 3C). The shift from 57 to 75 K is related to the stronger dipolar magnetic interactions between the 7.7 nm MNCs due to the decrease of their inter-particle distance.

For the binary superlattices, as no coalescence occurs, the size distribution is not modified. In this case, magnetic properties intermediate between the native and annealed maghemite films are observed: an increase of T_B_ (ΔT_B_ = 35 K) from 57 to 92 K (Figure 3D, green up triangles) and of the coercive field (ΔHc = 144 Oe) from 230 to 374 Oe (Figure 3E). This magnetic behavior is directly related to an increase of magnetic dipolar interactions related to the decrease in the inter-NPs distance after annealing.

If we compare the hysteresis loops for both annealed maghemite and binary superlattices, we can note some differences in the approach to the saturation that is slower for the annealed maghemite film compared to the binary superlattice while its coercivity is higher (Figure 3E). Monte-Carlo simulations by Kechrakos et al. give evidence that coalescence of NCs induces an increase of the coercivity due to a strong random dipolar fields generated in the sample. In addition, they show that the coalescence induces an increase of the remanence (Kechrakos and Trohidou, 2003). This is consistent with the strong dipolar magnetic interactions in the annealed inhomogeneous maghemite film compared to the binary superlattice. The presence of POMs in the binary superlattice constitutes a physical barrier between the MNCs preventing their coalescence during annealing while keeping their organization.

### Characterization of the Constitutive Elements

**Figure 4:**
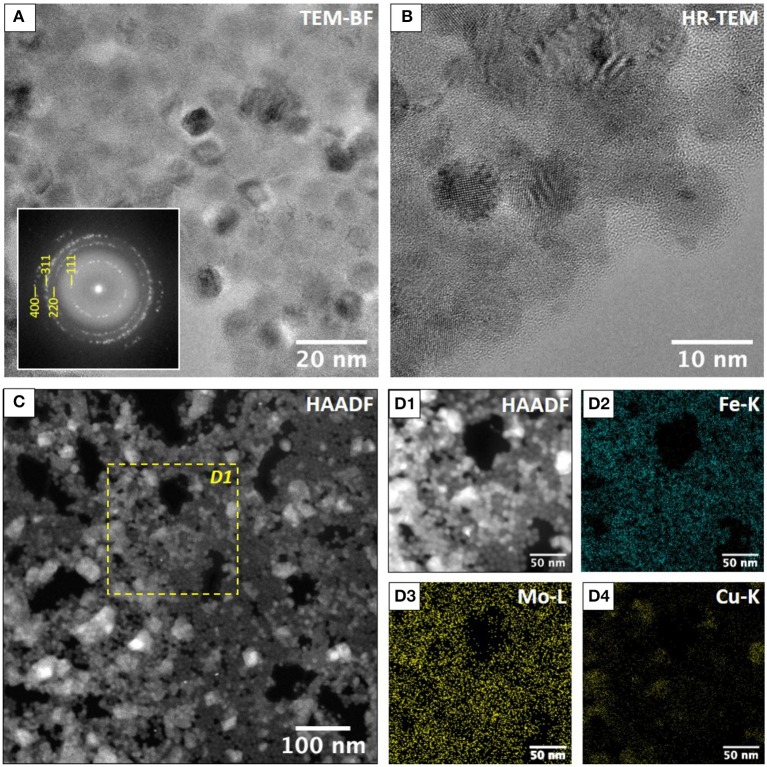
**(A)** TEM bright field image of maghemite single crystals. The corresponding Fast Fourier Transform in indexed using the cubic cell with *a* = 8.4 Å. **(B)** HR-TEM image showing the presence of maghemite single crystals embedded in an amorphous matrix. **(C)** STEM-HAADF of annealed g-Fe2O3 NCs/{Mo_132_} POMs binary superlattice 310°C under vacuum for 1 h. Dashed square corresponds to the STEM-XEDS elemental mapping shown in **(D1–D4)**. **(D1)** STEM-HAADF and STEM-XEDS elemental mapping of Fe **(D2)**, Mo **(D3)**, and Cu **(D4)**. **(D3)** Shows that the amorphous matrix contains Mo. Cu clusters revealed in **(D4)** are due to Copper diffusion on TEM grid during the annealing.

To further characterize each nano-object in the film after annealing, transmission electronic microscopies characterizations have been carried out for both samples. In order to preserve the microscopy copper grid, the annealing conditions were slightly modified: 310°C for 15 min under primary vacuum. The inter-NCs zones can be properly observed in the TEM images that show coalesced NCs for the reference sample (Supplementary Figure 2) while an inter-MNCs spacing is visible in the case of the annealed binary film with a matrix coating these NCs (Figures 4A,B). TEM and HRTEM characterizations confirmed that the MNCs have kept their integrity, size and shape, even after the drastic thermal treatment. To study the fate of the POMs after annealing, we performed STEM-HAADF (Figures 4C,D1) and STEM-XEDS (Figures 4D2–D4; Supplementary Figure 3) to determine the chemical mapping. On the energy filtered images is observed a homogeneous distribution of iron (Figure 4D2) and molybdenum (Figure 4D3). The coexistence of the Mo and Fe signals indicates that the non-coalescence of the MNCs is well induced by the presence of a Mo oxide matrix. Note that the STEM image and the chemical mapping revealed some copper contamination due to the partial degradation of the grid after annealing (Figure 4D4). However, the STEM technique is not accurate enough to precisely determine if the POMs have kept their integrity after annealing. To do so, a powder of DDA-Mo_132_ was treated independently by the same procedure as for the binary film (370°C, 1 h under vacuum) then characterized by infrared spectroscopy (IR) and X-ray powder diffraction (XRPD). According to the thermogravimetric analysis (Supplementary Figure 4A), an annealing at 370°C should totally desorb the DDA counter-cations surrounding the POMs. This is confirmed by the IR spectrum after annealing (Supplementary Figure 4B) in which the two bands at 2,852 and 2,923 cm^−1^ corresponding to the CH bonds stretching in the DDA counter cation disappeared. However, in the IR spectrum a drastic evolution of the bands between 500 and 1,000 cm^−1^ characteristic of the inorganic core of the DDA-Mo_132_ is also observed. This shows that the structure of the POM is no longer preserved under annealing. To determine the nature of the phase after annealing, a powder diffractogram was recorded and the MoO_3_ phase could be unambiguously determined (Supplementary Figure 4C). Under annealing, the DDA-Mo_132_ moves to the MoO_3_ oxide but still ensures the coating of the MNCs, which permits the non-coalescence of the MNCs and the preservation of their periodical arrangement.

### Limit of the Protective Effect: Annealing at 450°C

The protective effect of POMs in the film of MNCs shows a limit, as demonstrated by a similar study in which an annealing at 450°C for 1 h under vacuum was performed. As expected, the reference sample totally coalesces at this temperature (Supplementary Figure 5A). The corresponding blocking temperature is similar to the 370°C annealed reference (T_B_ = 216 K) but the shoulder at 75 K is not anymore observable (Supplementary Figure 5C, compare violet and blue curves), which means that the coalescence occurs not only at the surface but also in the volume of the film. Concerning the binary assembly, the SEM-FEG images show that at 450°C the surface layer of the binary film coalesced. However, an underlying layer of organized, non-coalesced MNCs persists even at this temperature (Supplementary Figure 5B). The corresponding magnetic properties are intermediate to the ones of the annealed binary superlattice at 370°C and the annealed maghemite film, with a T_B_ at 140 K. We emphasize that the ZFC curve is quite large around T_B_. In particular, a discrete shoulder can be observed at 100 K that could be explained by the presence of the under-layer of non-coalesced MNCs (Supplementary Figure 5C, dark green curve).

## Conclusion

In this article, we showed that the MNCs in binary assemblies made with oxo-clusters keep their morphology and organization under annealing. Up to around 370°C, the presence of the POMs in the octahedral sites of the MNCs lattice avoid the aggregation of the Fe_2_O_3_ NCs and preserves the periodicity even if the POM structure is destroyed. At this temperature, all the organic coating is desorbed, which permits to consider conductivity measurements in such systems. Moreover, the stability of the magnetic nanocrystals in the binary assembly until this temperature opens the way to the elaboration of new binary superlattices based on other nanocrystals such as Co, or CoPt, whose magnetic properties are very dependent of cristallinity (Lisiecki et al., 2007; Parker et al., 2007; Demortière and Petit, 2011; Andreazza et al., 2015).

## Data Availability Statement

All datasets generated for this study are included in the article/Supplementary Material.

## Author Contributions

RB synthesized the binary assemblies, did the annealing and characterized it. AG synthesized the binary assemblies and studied the stability of POMs under annealing. TA developed the method to make stable binary superlattices based on polyoxometalates and nanocrystals. A-TN did the synthesis of the maghemite nanocrystals. BB did the XRPD characterization. NM did the STEM-XEDS characterization. AP is at the origin of the collaboration between MONARIS and IPCM. CP is at the origin of the collaboration between MONARIS and IPCM. FV and CS managed the project and supervised the involved students.

### Conflict of Interest

The authors declare that the research was conducted in the absence of any commercial or financial relationships that could be construed as a potential conflict of interest.

## References

[B1] Alloyeau D. Prévot G. Le Bouar Y. Oikawa T. Langlois C. Loiseau A.. (2010). Ostwald ripening in nanoalloys: when thermodynamics drives a size-dependent particle composition. Phys. Rev. Lett. 105:255901. 10.1103/PhysRevLett.105.255901 21231603

[B2] Altantzis T. Yang Z. Bals S. Van Tendeloo G. Pileni M.-P. (2016). Thermal stability of CoAu _13_ binary nanoparticle superlattices under the electron beam. Chem. Mater. 28, 716–719. 10.1021/acs.chemmater.5b04898

[B3] Andreazza P. Pierron-Bohnes V. Tournus F. Andreazza-Vignolle C. Dupuis V. (2015). Structure and order in cobalt/platinum-type nanoalloys: from thin films to supported clusters. Surf. Sci. Rep. 70, 188–258. 10.1016/j.surfrep.2015.02.002

[B4] Bodnarchuk M. I. Erni R. Krumeich F. Kovalenko M. V. (2013). Binary superlattices from colloidal nanocrystals and giant polyoxometalate clusters. Nano Lett. 13, 1699–1705. 10.1021/nl4002475 23488858

[B5] Boles M. A. Talapin D. V. (2015). Many-body effects in nanocrystal superlattices: departure from sphere packing explains stability of binary phases. J. Am. Chem. Soc. 137, 4494–4502. 10.1021/jacs.5b00839 25773648

[B6] Breitwieser R. Auvray T. Volatron F. Salzemann C. Ngo A.-T. Albouy P.-A.. (2016). Binary superlattices from {Mo_132_} polyoxometalates and maghemite nanocrystals: long-range ordering and fine-tuning of dipole interactions. Small 12, 220–228. 10.1002/smll.201502127 26578032

[B7] Chen J. Dong A. Cai J. Ye X. Kang Y. Kikkawa J. M.. (2010). Collective dipolar interactions in self-assembled magnetic binary nanocrystal superlattice membranes. Nano Lett. 10, 5103–5108. 10.1021/nl103568q 21070007

[B8] Chen J. Ye X. Oh S. J. Kikkawa J. M. Kagan C. R. Murray C. B. (2013). Bistable magnetoresistance switching in exchange-coupled CoFe_2_O_4_–Fe_3_O_4_ binary nanocrystal superlattices by self-assembly and thermal annealing. ACS Nano 7, 1478–1486. 10.1021/nn3052617 23273052

[B9] Claridge S. A. Castleman A. W. Khanna S. N. Murray C. B. Sen A. Weiss P. S. (2009). Cluster-assembled materials. ACS Nano 3, 244–255. 10.1021/nn800820e 19236057

[B10] Demortière A. Petit C. (2011). CoPt magnetic nanocrystals in the A1/L1 _0_ transformation. J. Appl. Phys. 109:084344 10.1063/1.3575333

[B11] Dong A. Chen J. Vora P. M. Kikkawa J. M. Murray C. B. (2010). Binary nanocrystal superlattice membranes self-assembled at the liquid–air interface. Nature 466, 474–477. 10.1038/nature09188 20651688

[B12] Dong A. Chen J. Ye X. Kikkawa J. M. Murray C. B. (2011). Enhanced thermal stability and magnetic properties in NaCl-type FePt–MnO binary nanocrystal superlattices. J. Am. Chem. Soc. 133, 13296–13299. 10.1021/ja2057314 21800910

[B13] Dormann J. L. Spinu L. Tronc E. Jolivet J. P. Lucari F. D'Orazio F. (1998). Effect of interparticle interactions on the dynamical properties of γ-Fe_2_O_3_ nanoparticles. J. Magn. Magn. Mater. 183, L255–L260. 10.1016/S0304-8853(97)01123-2

[B14] Huang Z. Lu C. Dong B. Xu G. Ji C. Zhao K.. (2016). Chain stiffness regulates entropy-templated perfect mixing at single-nanoparticle level. Nanoscale 8, 1024–1032. 10.1039/C5NR06134B 26660086

[B15] Kang Y. Ye X. Chen J. Cai Y. Diaz R. E. Adzic R. R.. (2013a). Design of Pt–Pd binary superlattices exploiting shape effects and synergistic effects for oxygen reduction reactions. J. Am. Chem. Soc. 135, 42–45. 10.1021/ja3097527 23214936

[B16] Kang Y. Ye X. Chen J. Qi L. Diaz R. E. Doan-Nguyen V.. (2013b). Engineering catalytic contacts and thermal stability: gold/iron oxide binary nanocrystal superlattices for CO oxidation. J. Am. Chem. Soc. 135, 1499–1505. 10.1021/ja310427u 23294105

[B17] Kechrakos D. Trohidou K. N. (1998). Magnetic properties of dipolar interacting single-domain particles. Phys. Rev. B 58, 12169–12177. 10.1103/PhysRevB.58.12169

[B18] Kechrakos D. Trohidou K. N. (2003). Competition between dipolar and exchange interparticle interactions in magnetic nanoparticle films. J. Magn. Magn. Mater. 262, 107–110. 10.1016/S0304-8853(03)00029-5

[B19] Kiely C. J. Fink J. Brust M. Bethell D. Schiffrin D. J. (1998). Spontaneous ordering of bimodal ensembles of nanoscopic gold clusters. Nature 396, 444–446. 10.1038/24808

[B20] Klemmer T. J. Liu C. Shukla N. Wu X. W. Weller D. Tanase M. (2003). Combined reactions associated with L10 ordering. J. Magn. Magn. Mater. 266, 79–87. 10.1016/S0304-8853(03)00458-X

[B21] Ko D.-K. Urban J. J. Murray C. B. (2010). Carrier distribution and dynamics of nanocrystal solids doped with artificial atoms. Nano Lett. 10, 1842–1847. 10.1021/nl100571m 20411991

[B22] Künzle M. Eckert T. Beck T. (2016). Binary protein crystals for the assembly of inorganic nanoparticle superlattices. J. Am. Chem. Soc. 138, 12731–12734. 10.1021/jacs.6b07260 27617514

[B23] Lim S.-H. Lee T. Oh Y. Narayanan T. Sung B. J. Choi S.-M. (2017). Hierarchically self-assembled hexagonal honeycomb and kagome superlattices of binary 1D colloids. Nat. Commun. 8:360. 10.1038/s41467-017-00512-9 28842555 PMC5572454

[B24] Lisiecki I. Salzemann C. Parker D. Albouy P.-A. Pileni M.-P. (2007). Emergence of new collective properties of cobalt nanocrystals ordered in fcc supracrystals: I, structural investigation. J. Phys. Chem. C 111, 12625–12631. 10.1021/jp0718193

[B25] Lu F. Yager K. G. Zhang Y. Xin H. Gang O. (2015). Superlattices assembled through shape-induced directional binding. Nat. Commun. 6:6912. 10.1038/ncomms7912 25903309 PMC4423233

[B26] Mørup S. Hansen M. F. Frandsen C. (2010). Magnetic interactions between nanoparticles. Beilstein J. Nanotechnol. 1, 182–190. 10.3762/bjnano.1.22 21977409 PMC3045912

[B27] Müller A. Krickemeyer E. Bögge H. Schmidtmann M. Peters F. (1998). Organizational forms of matter: an inorganic super fullerene and keplerate based on molybdenum oxide. Angew. Chem. Int. Ed. Engl. 37, 3359–3363. 10.1002/(SICI)1521-3773(19981231)37:24<3359::AID-ANIE3359>3.0.CO;2-J 29711296

[B28] Ngo A.-T. Richardi J. Pileni M. P. (2013). Crack patterns in superlattices made of maghemite nanocrystals. Phys. Chem. Chem. Phys. 15:10666. 10.1039/c3cp50276g 23727907

[B29] Paik T. Diroll B. T. Kagan C. R. Murray C. B. (2015). Binary and ternary superlattices self-assembled from colloidal nanodisks and nanorods. J. Am. Chem. Soc. 137, 6662–6669. 10.1021/jacs.5b03234 25927895

[B30] Park J. An K. Hwang Y. Park J.-G. Noh H.-J. Kim J.-Y.. (2004). Ultra-large-scale syntheses of monodisperse nanocrystals. Nat. Mater. 3, 891–895. 10.1038/nmat1251 15568032

[B31] Parker D. Lisiecki I. Salzemann C. Pileni M.-P. (2007). Emergence of new collective properties of cobalt nanocrystals ordered in fcc supracrystals: II, magnetic investigation. J. Phys. Chem C 111, 12632–12638. 10.1021/jp071821u

[B32] Redl F. X. Cho K.-S. Murray C. B. O'Brien S. (2003). Three-dimensional binary superlattices of magnetic nanocrystals and semiconductor quantum dots. Nature 423, 968–971. 10.1038/nature01702 12827196

[B33] Rogach A. L. (2004). Binary superlattices of nanoparticles: self-assembly leads to Metamaterials. Angew. Chem. Int. Ed. 43, 148–149. 10.1002/anie.200301704 14695601

[B34] Talapin D. V. (2008). LEGO materials. ACS Nano 2, 1097–1100. 10.1021/nn8003179 19206324

[B35] Travesset A. (2017). Topological structure prediction in binary nanoparticle superlattices. Soft Matter 13, 147–157. 10.1039/C6SM00713A 27156535

[B36] Udayabhaskararao T. Altantzis T. Houben L. Coronado-Puchau M. Langer J. Popovitz-Biro R.. (2017). Tunable porous nanoallotropes prepared by post-assembly etching of binary nanoparticle superlattices. Science 358, 514–518. 10.1126/science.aan6046 29074773

[B37] Urban J. J. Talapin D. V. Shevchenko E. V. Kagan C. R. Murray C. B. (2007). Synergism in binary nanocrystal superlattices leads to enhanced p-type conductivity in self-assembled PbTe/Ag2Te thin films. Nat. Mater. 6, 115–121. 10.1038/nmat1826 17237786

[B38] Vanmaekelbergh D. (2011). Self-assembly of colloidal nanocrystals as route to novel classes of nanostructured materials. Nano Today 6, 419–437. 10.1016/j.nantod.2011.06.005

[B39] Wei J. Schaeffer N. Pileni M.-P. (2015). Ligand exchange governs the crystal structures in binary nanocrystal superlattices. J. Am. Chem. Soc. 137, 14773–14784. 10.1021/jacs.5b09959 26549642

[B40] Wu Y. Li S. Gogotsi N. Zhao T. Fleury B. Kagan C. R. (2017). Directional carrier transfer in strongly coupled binary nanocrystal superlattice films formed by assembly and *in situ* ligand exchange at a liquid–air interface. J. Phys. Chem. C 121, 4146–4157. 10.1021/acs.jpcc.6b12327

[B41] Yang Z. Wei J. Pileni M.-P. (2015). Metal–metal binary nanoparticle superlattices: a case study of mixing Co and Ag nanoparticles. Chem. Mater. 27, 2152–2157. 10.1021/acs.chemmater.5b00123

[B42] Ye X. Chen J. Diroll B. T. Murray C. B. (2013a). Tunable plasmonic coupling in self-assembled binary nanocrystal superlattices studied by correlated optical microspectrophotometry and electron microscopy. Nano Lett. 13, 1291–1297. 10.1021/nl400052w 23418862

[B43] Ye X. Millan J. A. Engel M. Chen J. Diroll B. T. Glotzer S. C.. (2013b). Shape alloys of nanorods and nanospheres from self-assembly. Nano Lett. 13, 4980–4988. 10.1021/nl403149u 24044735

[B44] Ye X. Zhu C. Ercius P. Raja S. N. He B. Jones M. R.. (2015). Structural diversity in binary superlattices self-assembled from polymer-grafted nanocrystals. Nat. Commun. 6:10052. 10.1038/ncomms10052 26628256 PMC4686769

[B45] Zeng H. Li J. Liu J. P. Wang Z. L. Sun S. (2002). Exchange-coupled nanocomposite magnets by nanoparticle self-assembly. Nature 420, 395–398. 10.1038/nature01208 12459779

