# Supplementary material for: Protective Effect of Polyoxometalates in {Mo132}/Maghemite Binary Superlattices Under Annealing
Source: Front Chem. 2019 Nov 29;7:830. doi: 10.3389/fchem.2019.00830 (PMC6895896; doi:10.3389/fchem.2019.00830)

Supplementary Material

Protective effect of polyoxometalates in {Mo_132_}/Maghemite binary superlattices under annealing

R. Breitwieser,^1,2^ A. Garnier,^1,2^ T. Auvray,^1,2^ A.-T. Ngo,^1^ B. Baptiste,^3^ N. Menguy^3^, A. Proust,^2^ C. Petit,^1^ F. Volatron,^2*^ C. Salzemann^1*^

^1^Laboratory MONARIS, Sorbonne Université, CNRS UMR 8233, 75252 Paris Cedex 05, France

^2^Laboratory IPCM, Sorbonne Université, CNRS UMR 8232, 75252 Paris Cedex 05, France

^3^Laboratory IMPMC, Sorbonne Université, CNRS UMR 7590, 75252 Paris Cedex 05, France

**Supplementary Figure 1.** FEG-SEM images and the corresponding plot profiles for the native and annealed (at 370°C for 1h) samples: (A) native and (B) annealed Fe_2_O_3_ films ; (C) native and (D) annealed binary superlattices.


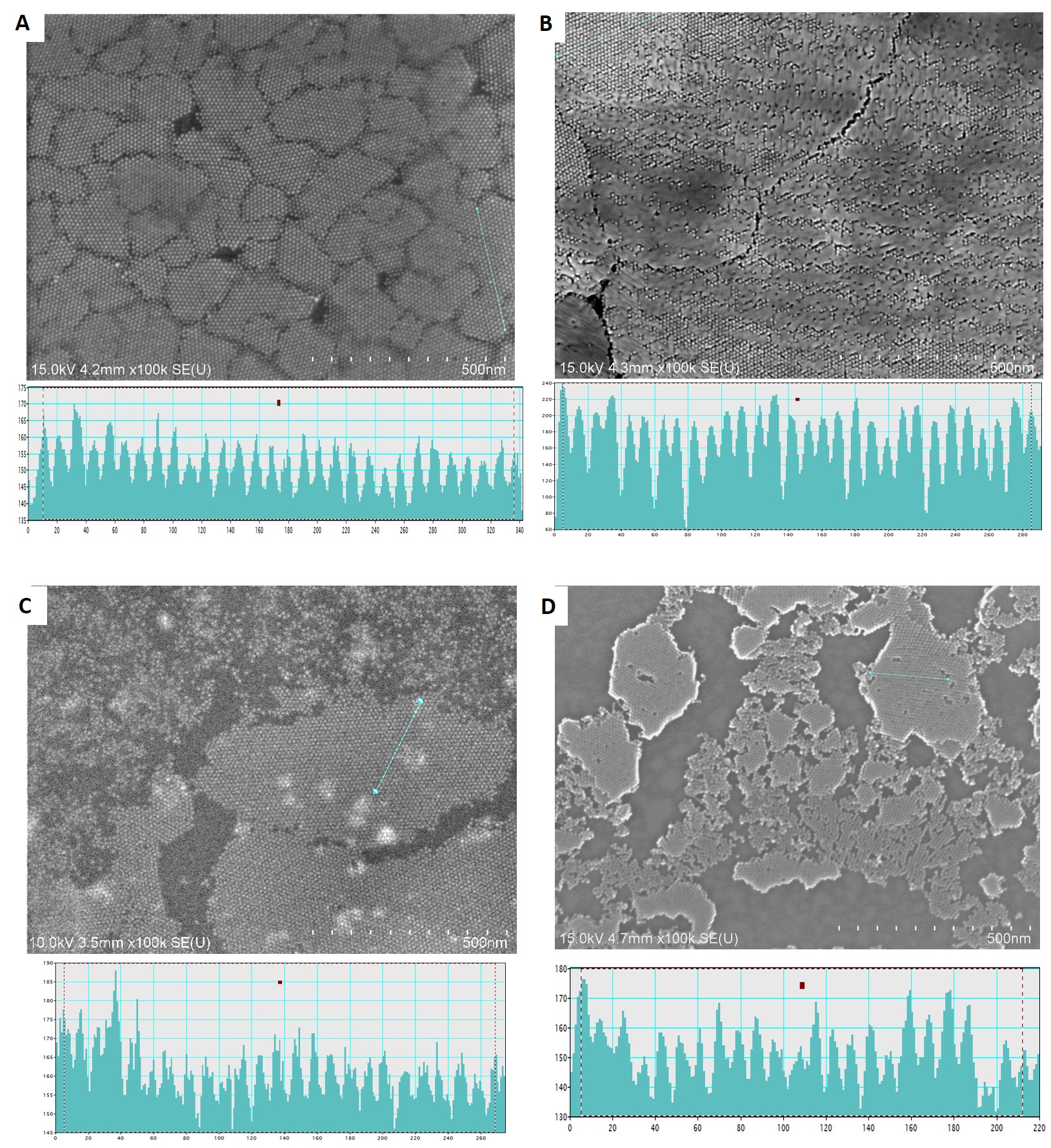


**Supplementary Figure 2.** TEM image of the Fe_2_O_3_ film annealed at 310°C for 15min.

**
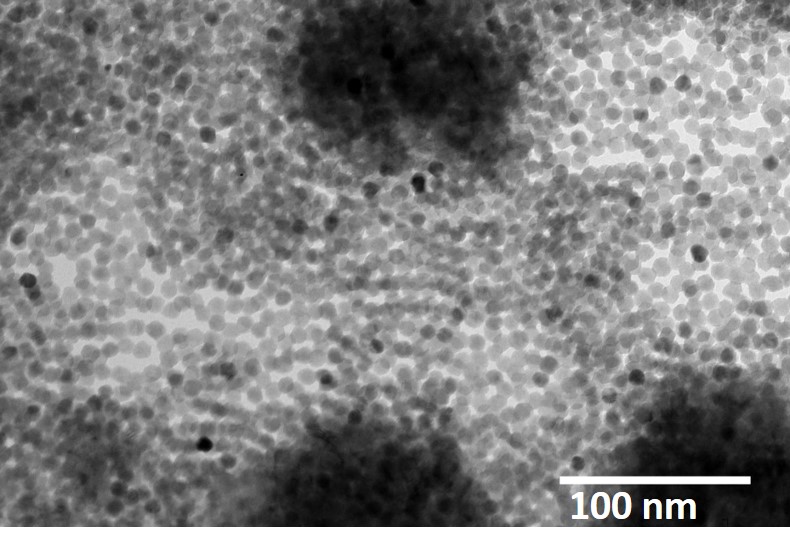
**

**Supplementary Figure 3.** Energy dispersive spectrum from STEM analysis corresponding to the binary superlattices annealed at 310°C for 15 min.

**
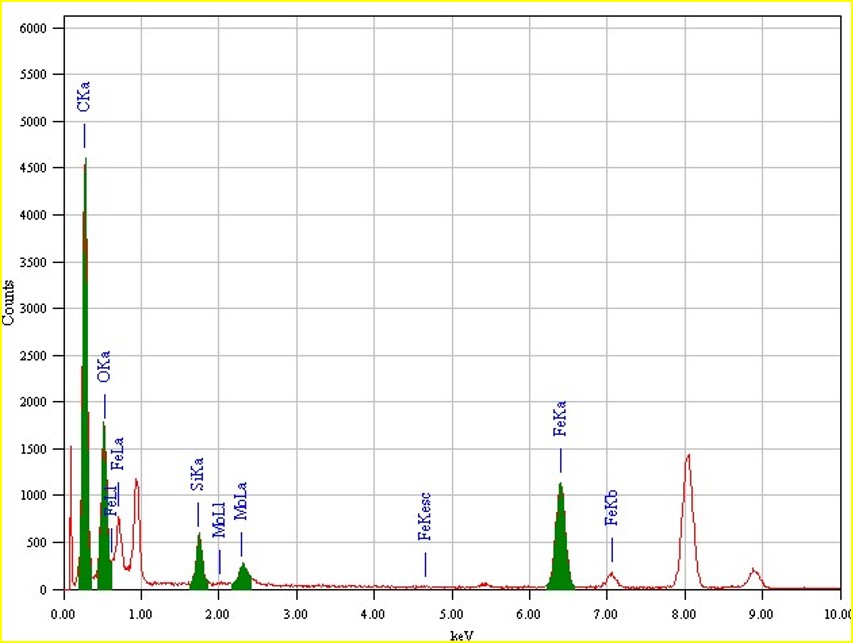
**

**Supplementary Figure 4.** Analysis of the annealing effect on a DDA-Mo_132_ powder (A) TGA measurements under N_2_ : weight loss in (%) as a function of temperature (ramp 5°C /min) (B) IR spectra in a KBr pellet of the powder before (red) and after (blue) annealing (370°C, 1h under vacuum) (C) X-ray powder diffractogram of the powder after annealing (370°C, 1h under vacuum).

**Supplementary Figure 5.** FEG-SEM images of a (A) γ-Fe_2_O_3_ film and (B) γ-Fe_2_O_3_/Mo_132_ binary superlattice on HOPG after annealing at 450°C for one hour under vacuum. The corresponding temperature-dependence (FC and ZFC measured with in-plane field of 100 Oe) of annealed γ-Fe_2_O_3_ (violet ⊗) and γ-Fe_2_O_3_/{Mo_132_} binary film (dark green stars).


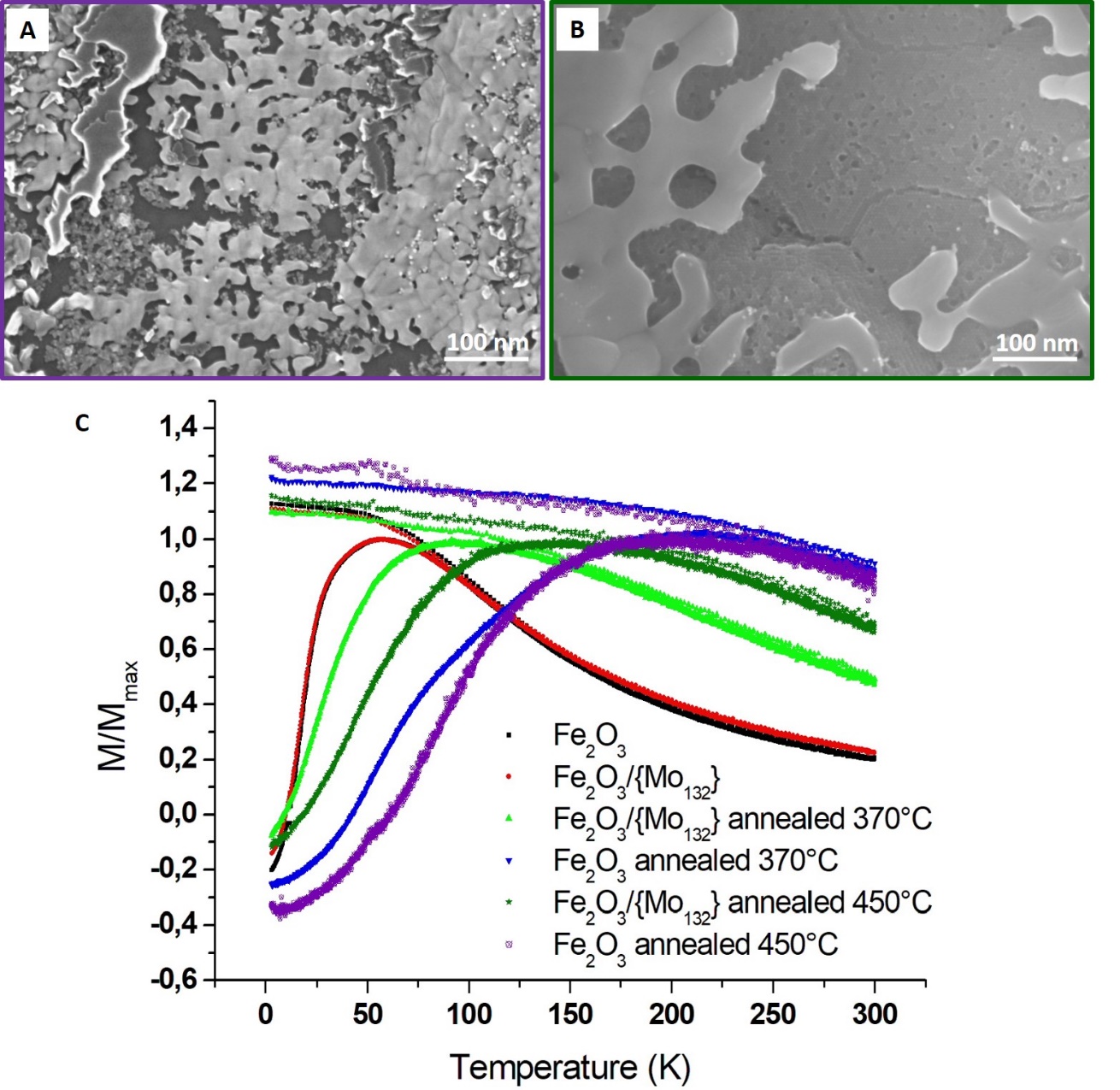

Supplement: Supplementary file 1 [file Table_1.DOCX]
